# Research Trends and Hotspots on Herpes Zoster: A 10-Year Bibliometric Analysis (2012–2021)

**DOI:** 10.3389/fmed.2022.850762

**Published:** 2022-04-26

**Authors:** Jian Zhang, Xu Han, Diansan Su, Xiyao Gu, Weifeng Yu

**Affiliations:** ^1^NMPA Key Laboratory for Research and Evaluation of Narcotic and Psychotropic Drugs, Jiangsu Province Key Laboratory of Anesthesiology, Jiangsu Province Key laboratory of Anesthesia and Analgesia, Xuzhou Medical University, Xuzhou, China; ^2^Department of Anesthesiology, Department of Radiology, Renji Hospital, Shanghai Jiao Tong University School of Medicine, Shanghai, China

**Keywords:** herpes zoster, citespace, bibliometric analysis, keywords co-occurrence and burst keywords, cited reference

## Abstract

**Purpose:**

Herpes zoster infection, with its considerable burden to individuals and society, remains a challenge around the world. However, to the knowledge of the authors, little bibliometric quantitative or qualitative analysis has been carried out to evaluate herpes zoster research. This study aimed to use a bibliometric analysis to evaluate current publication trends and hotspots on herpes zoster research worldwide, in order to advance research in this field.

**Methods:**

Relevant publications from January 2012 to December 2021 were collected from the Web of Science Core Collection database. Citespace (V5.8.R3) was used to analyze the research points, including publication countries, institutions and authors, cited author, cited reference and their clustering, and keyword co-occurrence, and burst keyword to acquire research trends and hotspots.

**Results:**

A total of 9,259 publications were obtained, with a steady increase in the number of annual publications during the decade. Articles were the main type of publication. The United States is the leading country in this research, and the University of Colorado has the highest influence in this field. Oxman is the most representative author, with a main research interest in herpes zoster vaccines. The top five cited authors' publications focused on herpes zoster vaccines, molecular mechanisms, and postherpetic neuralgia. A co-citation map resulted 19 main clusters, and revealed that vaccines, postherpetic neuralgia, treatments, varicella zoster virus and its mechanisms, and epidemiology of herpes zoster were the current research focus after clustering co-cited publications. Human herpesviruses, antiviral prophylaxis, rheumatoid arthritis, recombinant zoster vaccine, varicella vaccination and postherpetic neuralgia were the top clusters after co-occurrence keywords analysis. Moreover, burst keywords detection showed that the subunit vaccine was the new hotspot in the field of herpes zoster.

**Conclusion:**

This bibliometric study defined the overall prospects in the field of herpes zoster and provided valuable instruction for the ongoing research. The keyword “subunit vaccine” indicated that a vaccine for herpes zoster prevention was the hotspot. Efforts to prevent varicella zoster virus infection will be essential to improve herpes zoster outcomes.

## Introduction

Herpes zoster infection has a high incidence especially among the elderly, with 20–30% of the population affected at any time (1). A milestone in herpes zoster research was accomplished by Hope-Simpson in 1964, who observed the infection of chickenpox and shingles in people living on a small Scottish island, and hypothesized that herpes zoster is caused by a reactivation of the chickenpox virus (2). It is clear now that herpes zoster is caused by the reactivation of varicella zoster virus (VZV). Primary infection with VZV leads to varicella, during which VZV is latent in the ganglion neurons. Individuals with lower immunity have a high risk of reactivated VZV, and subsequent herpes zoster (3–5).

Herpes zoster infection usually manifests as a blistered, painful skin rash with several complications, causing a considerable burden to patients, society, and the country (6, 7). The most common and difficult to tolerate symptom is postherpetic neuralgia, which can cause severe chronic pain and limitations in activities (8–10). It can also lead to meningitis or meningoencephalitis, cerebellitis, isolated cranial nerve palsies that produce ophthalmoplegia or the Ramsay Hunt syndrome, multiple cranial nerve palsies (polyneuritis cranialis), vasculopathy, myelopathy, and various inflammatory disorders of the eye and other organs (4, 5). Efforts have been made in daily clinical practice to alleviate patient suffering due to herpes zoster, with early treatments like the application of antiviral drugs such as acyclovir, or analgesic drugs such as carbamazepine and pregabalin combined with neurotrophic drugs, achieved good efficacy. For drug-refractory cases, interventional, physical, and psychological therapies are available (6, 11). Pulsed radiofrequency therapy has gained popularity in recent years (7). Because eradication of the virus itself is difficult, prevention of infection is an attractive route for researchers. A vaccine has been available for over 50, and a pediatric vaccine is now undergoing clinical trials (12–14). However, the incidence of herpes zoster infection remains high, and patients worldwide still have a lower life quality due to the infection (15).

Bibliometric analysis refers to the quantitative analysis of publications using mathematical and statistical methods. This comprehensive system of mathematics, statistics and philology focuses on quantification, and is used by researchers to quickly identify current trends in their field of research (16–18). Citespace, developed by Chaomei, is a commonly used software for visual analysis of a large number of documents in bibliometric analysis (19–21). However, few quantitative analyses have been reported on herpes zoster, and little is known about current hot topics or trends in this area via analysis and prediction. In current study, we analyzed publications relating to herpes zoster to evaluate research trends and identify hot spots. Herein, we briefly visualize and interpret our results on herpes zoster research, and discuss the trends in this field over the next few years.

## Materials and Methods

### Data Source and Search Strategy

A comprehensive search for publications related to herpes zoster was conducted through the Web of Science Core Collection (WoSCC) database, Science Citation Index Expanded (SCI-E) Edition. The following search strategy was applied: [zoster] OR [shingles] OR [zona] from January 1, 2012 to December 31, 2021. Two investigators (Jian Zhang and Xu Han) reviewed all the publications and screened the publications unrelated to the topic. No restrictions were applied in terms of publication type, language, or data category. All searches and data downloads were completed in a single day, January 5, 2022, to minimize deviations caused by frequent updates to the database.

### Data Processing

The publications were screened and their characteristics, including titles, authors, keywords, country, institution and cited references, were recorded and converted to plain text format, and then imported to CiteSpace V5.8.R3, 64 bit (Drexel University, Philadelphia, PA, USA) for further bibliometric analysis.

### Bibliometric Analysis

All literature characteristics were described, including countries/regions, institutions, authors, cited authors, clustered networks of co-cited references and keywords, and keywords with the strongest citation bursts. CiteSpace software was used to visualize the bibliometric data. Data were imported into CiteSpace and an integrated network was created to simulate the knowledge structure within the domain. Each color of the tree rings in the node represents series of publication year and the thickness of the rings represents the number of publications/citations obtained from the data inputted. The purple circle represents the nodes with high centrality, with the thickness of the purple part indicating the strength of the centrality. Centrality is a metric associated with the transformative potential of a scientific contribution. The centrality of a node quantifies the importance of the node's position in a network. Betweenness centrality metric measures the percentage of the number of shortest paths in a network to which a given node belongs. High centrality could be found in hubs where nodes are strongly connected to other nodes, and nodes in the middle of two classes of nodes. Keyword frequency over 50 and top 50 keywords occurrences each year was set as the threshold to generate clusters and figures. Burst detection is a computational technique for detecting sudden changes in events and other types of information that reflect the breakthrough achievement.

## Results

### Bibliometric Analysis of Publication Outputs

**Figure 1:**
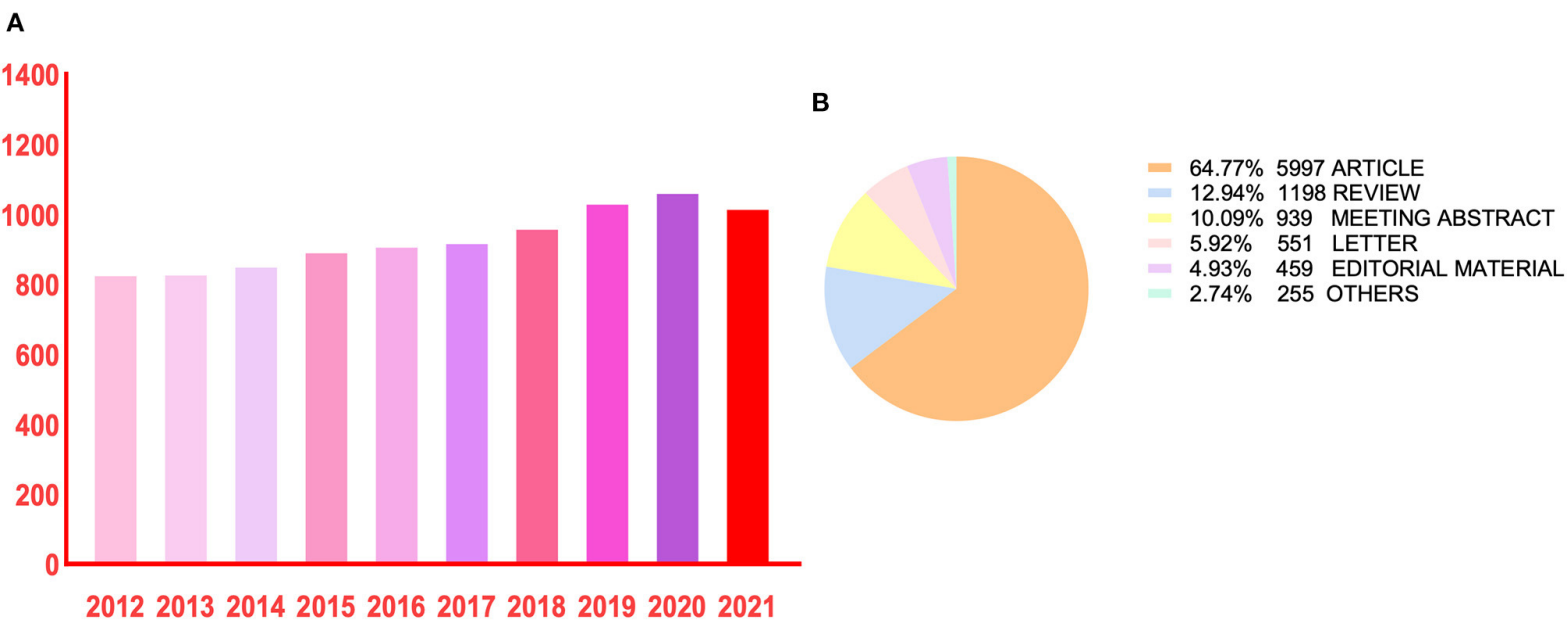
Bibliometric analysis of publication outputs. 9,259 publications were included from 2012 to 2021. **(A)** The number of annual publications on herpes zoster. **(B)** The distribution of article type.

A total of 9,259 publications were identified in the bibliometric analysis. The number of publications relating to herpes zoster increased every year, with the exception of a slight decrease in 2021 compared with 2020 (Figure 1A). The difference between 2012 and 2020 (823 vs. 1,058 publications, respectively) suggests that herpes zoster is gaining increasing attention around the world.

Nineteen publication types concentrating on herpes zoster were identified (Figure 1B). Articles were for the most common publication type (5,997, 64.77%), followed by reviews (1,198, 12.94%) and meeting abstracts (939, 10.09%). In total, 135 countries/regions were involved, with the United States (US) contributing more than one-third of the total publications (3,186, 34.4%), nearly five-times higher than Germany (717, 7.7%) or Chinese mainland (702, 7.6%) (Table 1). The top five productive institutions included the University of Colorado, with others distributed around the U.S. and United Kingdom contributing a total of 675 publications (Table 1).

**Table 1:** Top 10 countries/region and institutions in terms of publications for herpes zoster.

**Ranking**	**Country/region**	**Publications**	**Institution**	**Publications**
1	USA	3,186	University of Colorado	214
2	Germany	717	GlaxoSmithKline	130
3	Chinese mainland	702	Oregon Health and Science University	116
4	Japan	657	Stanford University	108
5	United Kingdom	607	Disease Control and prevention	107
6	Italy	461	Mayo clinic	106
7	Canada	424	University of Toronto	103
8	France	410	University of Pennsylvania	97
9	South Korea	404	University of *Alabama*- *Birmingham*	97
10	Belgium	374	Harvard Medical School	90

### Collaborating Countries/Regions and Institutions on Herpes Zoster Research

**Figure 2:**
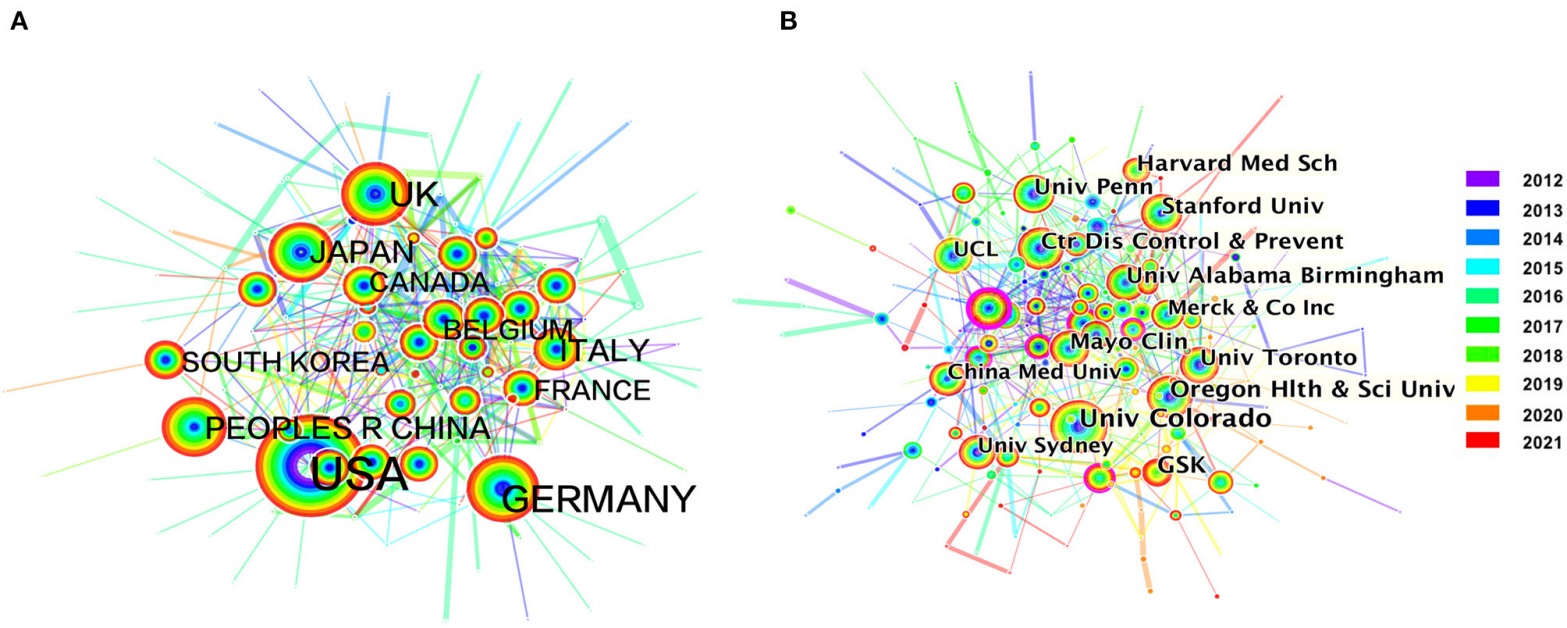
The network map of collaborating countries/regions **(A)** and institutions **(B)** in herpes zoster research.

National/regional cooperation analysis is relevant for the published countries or regions, reflecting the cooperation between countries/regions in a certain research field. After conducting this analysis via CiteSpace, 149 nodes and 422 links were detected, which represent 149 countries/regions contributing to herpes zoster research. The U.S. is clearly defined as the center of national cooperation and exchange with other countries, followed by Germany and the United Kingdom (Figure 2A, Supplementary Figure 1). Regional clustering was also apparent around the world, with Asian countries including the Peoples Republic of China, Japan and South Korea displaying a prodigious exchange. The assessment of centrality reflects the influence and importance of the nodes in the network. The U.S. undoubtly had the highest influence (0.21), followed by the Germany (0.14) and the United Kingdom (0.14) (Table 2).

**Table 2:** Top 10 countries/region and institutions in terms of centrality for herpes zoster.

**Ranking**	**Country/region**	**Centrality**	**Institution**	**Centrality**
1	USA	0.21	Charite	0.06
2	Germany	0.14	University of Michigan	0.05
3	United Kingdom	0.14	University of São Paulo	0.05
4	France	0.14	University of Maryland	0.05
5	Canada	0.13	National Institute of Allergy and Infectious Diseases	0.05
6	Belgium	0.11	University of California, San Francisco	0.04
7	Netherlands	0.08	Johns Hopkins University	0.04
8	Australia	0.07	Pfizer Inc.	0.04
9	Spain	0.07	Harvard University	0.04
10	Italy	0.06	McGill University	0.04

We also conducted an analysis of institutional cooperation. Generating an institution map resulted in 1,176 nodes and 3,881 links, with a density (0.0056) that suggests that the research in each institution is relatively scattered (Figure 2B, Supplemental Figure 2). As before, institutions in the U.S. had the highest number of publications, except for GlaxoSmithKline (GSK) in the United Kingdom. The University of Colorado contributed the most publications (214), followed by GSK (130), and Oregon Health and Science University (116). In terms of centrality, the top three institutions were the Charite (0.06), the University of Michigan (0.05), and the University of São Paulo (0.05) (Table 2). Taken together, these analyses suggest that more academic exchanges are needed.

### Analysis of Author and Co-cited Author

**Figure 3:**
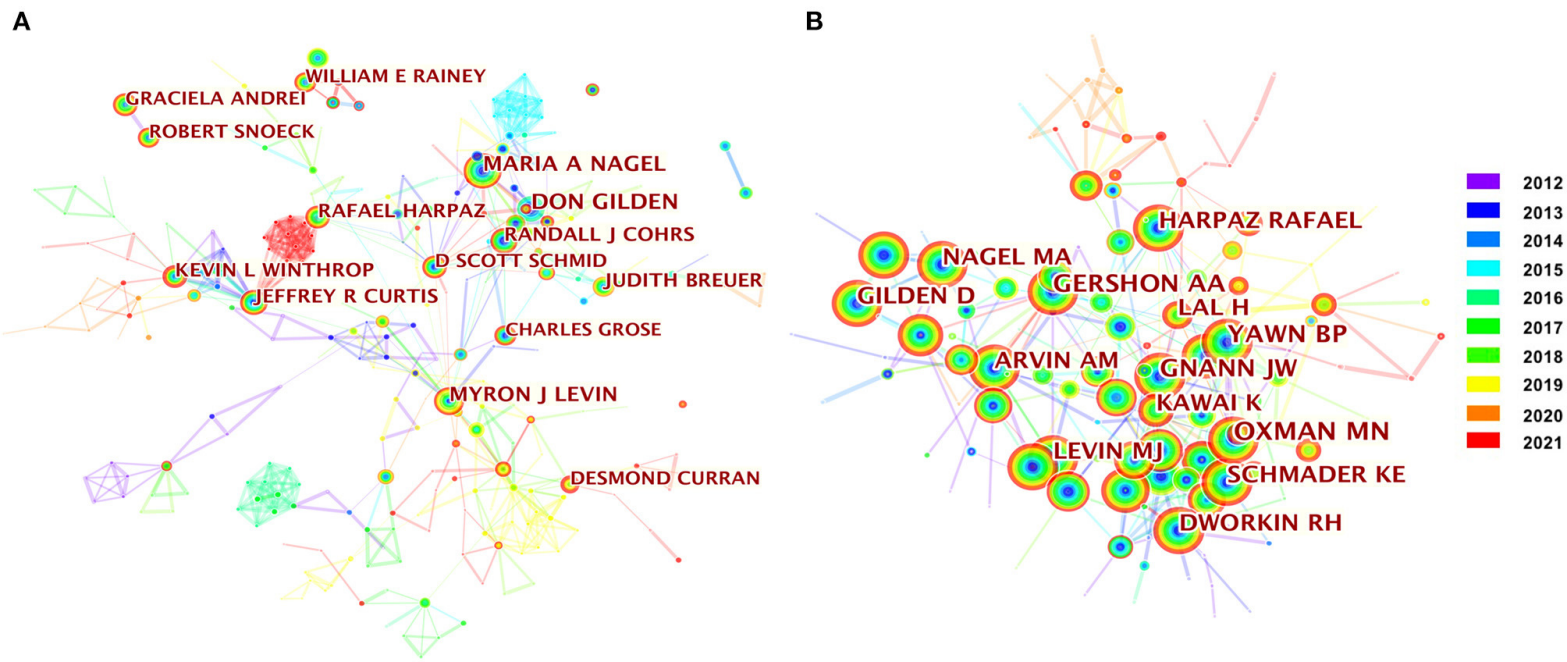
The network map of authors **(A)** and the co-cited authors **(B)** map in herpes zoster research.

Citespace identified 2,716 nodes and 8,126 links for author collaboration analysis, which indicated that 2,716 authors contributed to 9,259 publications, and their potential cooperation (Figure 3A, Supplementary Figure 3). The low density (0.0053) suggest that the authors in herpes zoster research is relatively dispersed. Among them, the three most productive authors were Gilden (80), followed by Nagel (65), and Levin (58). In terms of centrality, the top three authors were Levin (0.04), Harpaz (0.04), and Schmid (0.04) (Table 3), whose research interests include clinical research on herpes zoster vaccines. Their work includes the clinical characteristics of herpes zoster after varicella vaccine in children, and the use of the herpes zoster subunit vaccine in the elderly (22–26).

**Table 3:** Top 10 active authors in terms of publications and centrality for herpes zoster.

**Ranking**	**Publications**	**Author**	**Centrality**	**Author**
1	80	Don Gilden	0.04	Myron J. Levin
2	65	Maria A. Nagel	0.04	Rafael Harpaz
3	58	Myron J Levin	0.04	D. Scott Schmid
4	44	Rafael Harpaz	0.03	Barbara P. Yawn
5	43	Kevin L. Winthrop	0.03	Stephen Hall
6	42	Jeffrey R. Curtis	0.02	Don Gilden
7	41	Judith Breuer	0.02	Anthony L Cunningham
8	40	Randall J. Cohrs	0.01	Maria A. Nagels
9	38	William E. Rainey	0.01	Kevin L. Winthrop
10	38	Graciela Andrei	0.01	Jeffrey R. Curtis

A co-cited author analysis is an analysis of the authors of a reference, with clustering of the authors with highly similar research interests. In total, 789 nodes and 1,838 links were identified via CiteSpace (Figure 3B, Supplementary Figure 4). Oxman had the top rank for citation counts (830), with studies predominantly focused on herpes zoster vaccines (27–31). Among the rest highly co-cited authors, Gershon had reported widely on the virus and its molecular mechanisms, and provided the first solid evidence for vaccination against varicella disease (32–36) (Table 4).

**Table 4:** Top 10 co-cited authors in herpes zoster research in terms of co-citation counts and centrality.

**Ranking**	**Co-citation counts**	**Cited author**	**Centrality**	**Cited author**
1	830	Oxman MN	0.13	Follett KA
2	591	Yawn BP	0.12	Plaha P
3	540	Gershon AA	0.1	Singh JA
4	502	Gnann JW	0.1	Khan S
5	453	Levin MJ	0.09	Coplan PM
6	452	Arvin AM	0.09	Hayward AR
7	448	Dworkin RH	0.08	Winthrop KL
8	442	Lal H	0.08	Breuer J
9	434	Schmader KE	0.08	Seward JF
10	429	Nagel MA	0.07	Arvin AM

### Clustering of Co-cited Publications

**Figure 4:**
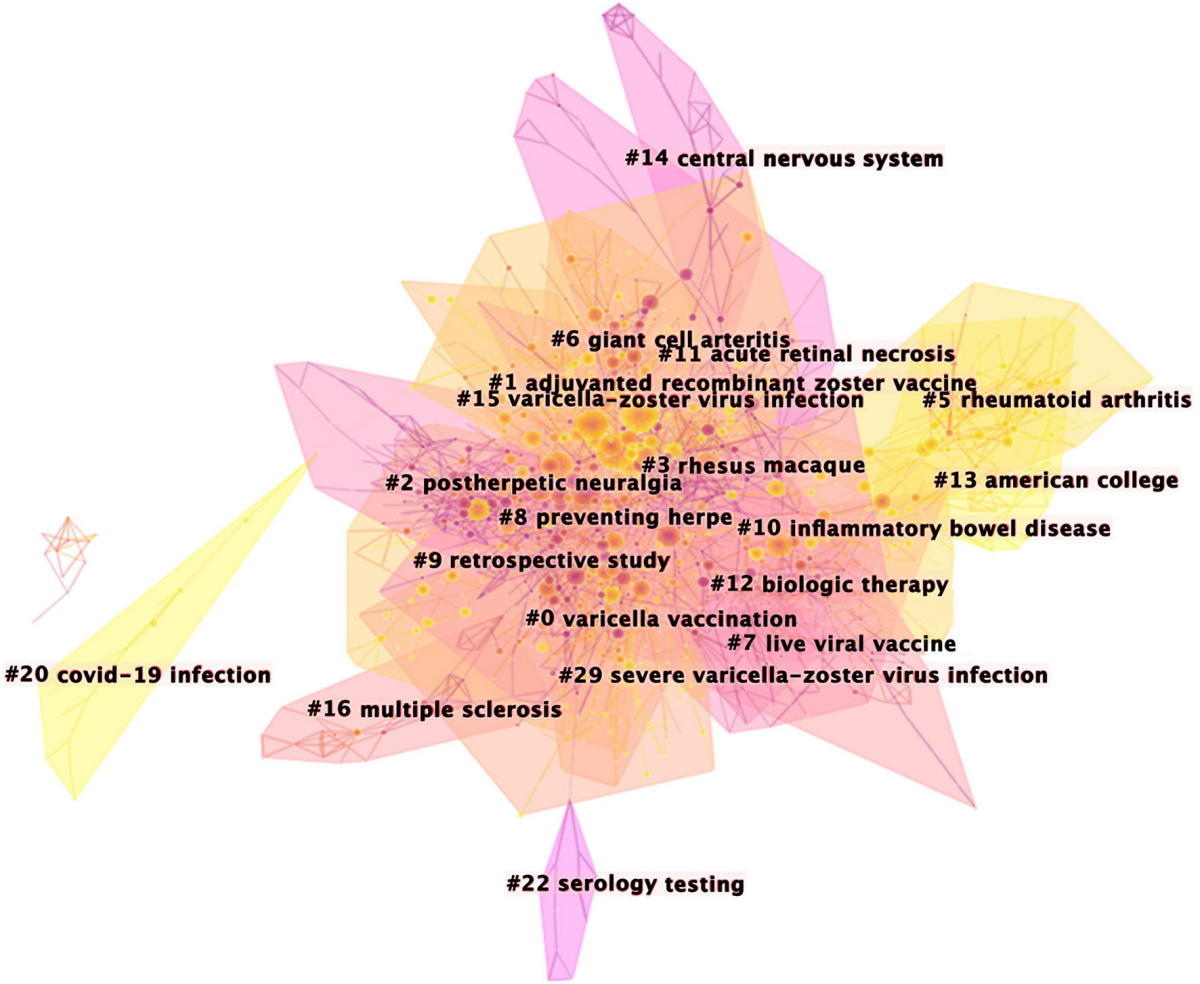
The clustered network map of co-cited references on herpes zoster.

**Figure 5:**
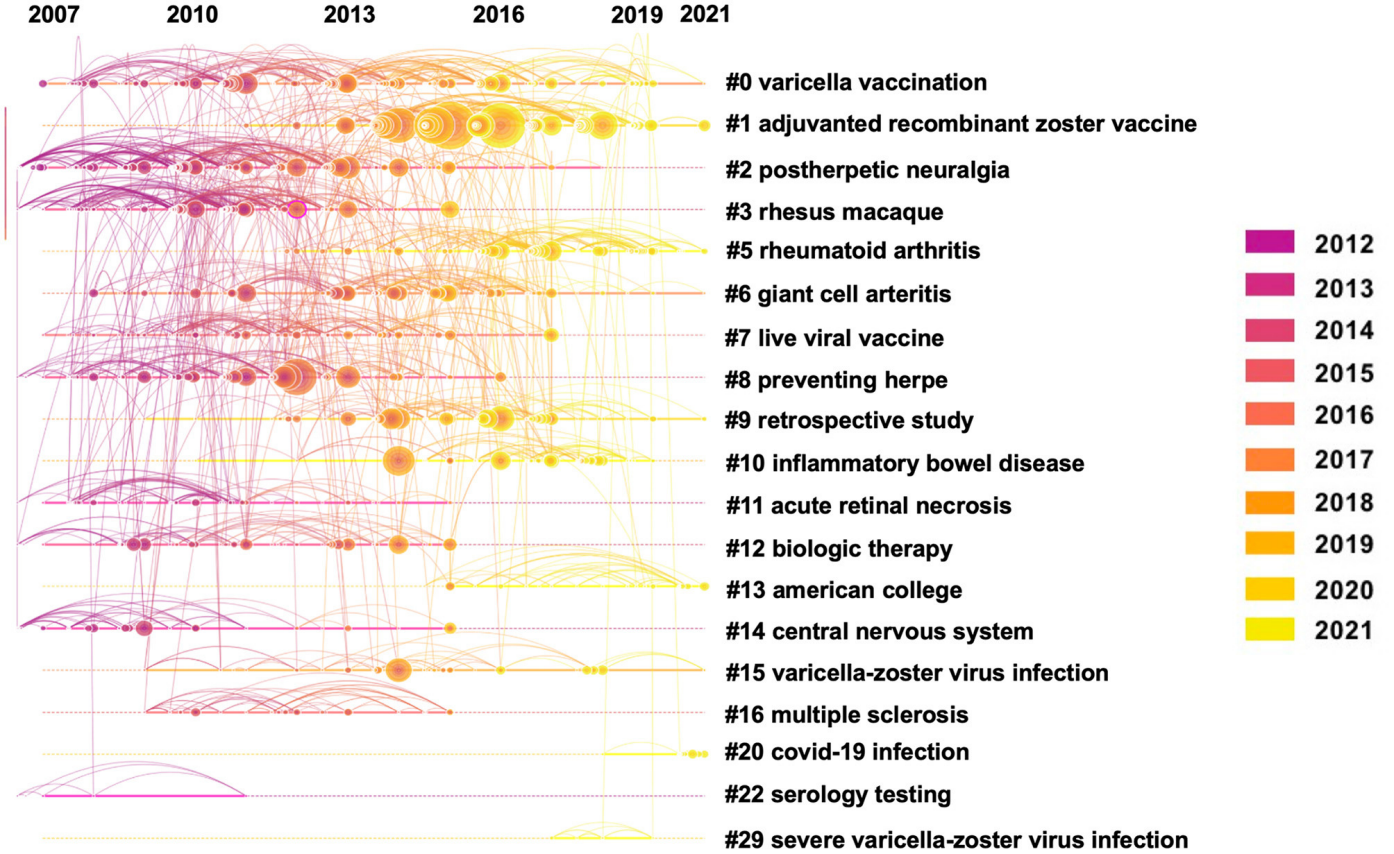
The timeline view of co-cited references clusters with their cluster-labels on the right.

Co-cited publications reveal the relevance and knowledge base of the publications (37). Over half of the top 10 co-cited publications related to herpes zoster vaccine (Table 5). With 351 citations, the most cited publication was the clinical trial by Himal and colleagues published in the New England Journal of Medicine in 2015 (38). This was a randomized, placebo-controlled, phase 3 study in 18 countries to evaluate the efficacy and safety of a herpes zoster subunit in older adults (≥50 years of age), stratified according to age group (50 to 59, 60 to 69, and ≥70 years). Participants received two intramuscular doses of the vaccine or placebo, 2 months apart. The vaccine reduced the risk of herpes zoster in adults who were 50 years of age or older. Vaccine efficacy in adults who were 70 years of age or older was similar to that in the other two age groups. Further clustering these references resulted in 1,506 nodes, 2,841 links, and 19 main clusters (Figure 4), and clearly showed the timeline for each cluster label (Figure 5). The position of the nodes in these clusters suggests that the predominant research focus in herpes zoster involves vaccines, postherpetic neuralgia, treatments, the VZV and its mechanism, and the epidemiology of herpes zoster infection.

**Table 5:** Top 10 co-cited references related to herpes zoster in terms of cocitations.

**Ranking**	**Co-citation** **counts**	**Cited reference**	**References**
1	351	Efficacy of an Adjuvanted Herpes Zoster Subunit Vaccine in Older Adults	(38)
2	318	Efficacy of the Herpes Zoster Subunit Vaccine in Adults 70 Years of Age or Older	(24)
3	169	Systematic review of incidence and complications of herpes zoster: toward a global perspective	(39)
4	153	Recommendations of the Advisory Committee on Immunization Practices for Use of Herpes Zoster Vaccines	(40)
5	142	Long-Term Persistence of Zoster Vaccine Efficacy	(41)
6	138	Efficacy, Safety, and Tolerability of Herpes Zoster Vaccine in Persons Aged 50–59 Years	(25)
7	104	2013 IDSA Clinical Practice Guideline for Vaccination of the Immunocompromised Host	(42)
8	92	Links between herpes zoster incidence and childhood varicella vaccination	(43)
9	90	A systematic review and meta-analysis of risk factors for postherpetic neuralgia	(44)
10	90	Association Between Vaccination for Herpes Zoster and Risk of Herpes Zoster Infection Among Older Patients With Selected Immune-Mediated Diseases	(45)

### Cluster Analysis of Keyword Co-occurrence on Herpes Zoster Hotspots and Burst Keywords Detection

**Figure 6:**
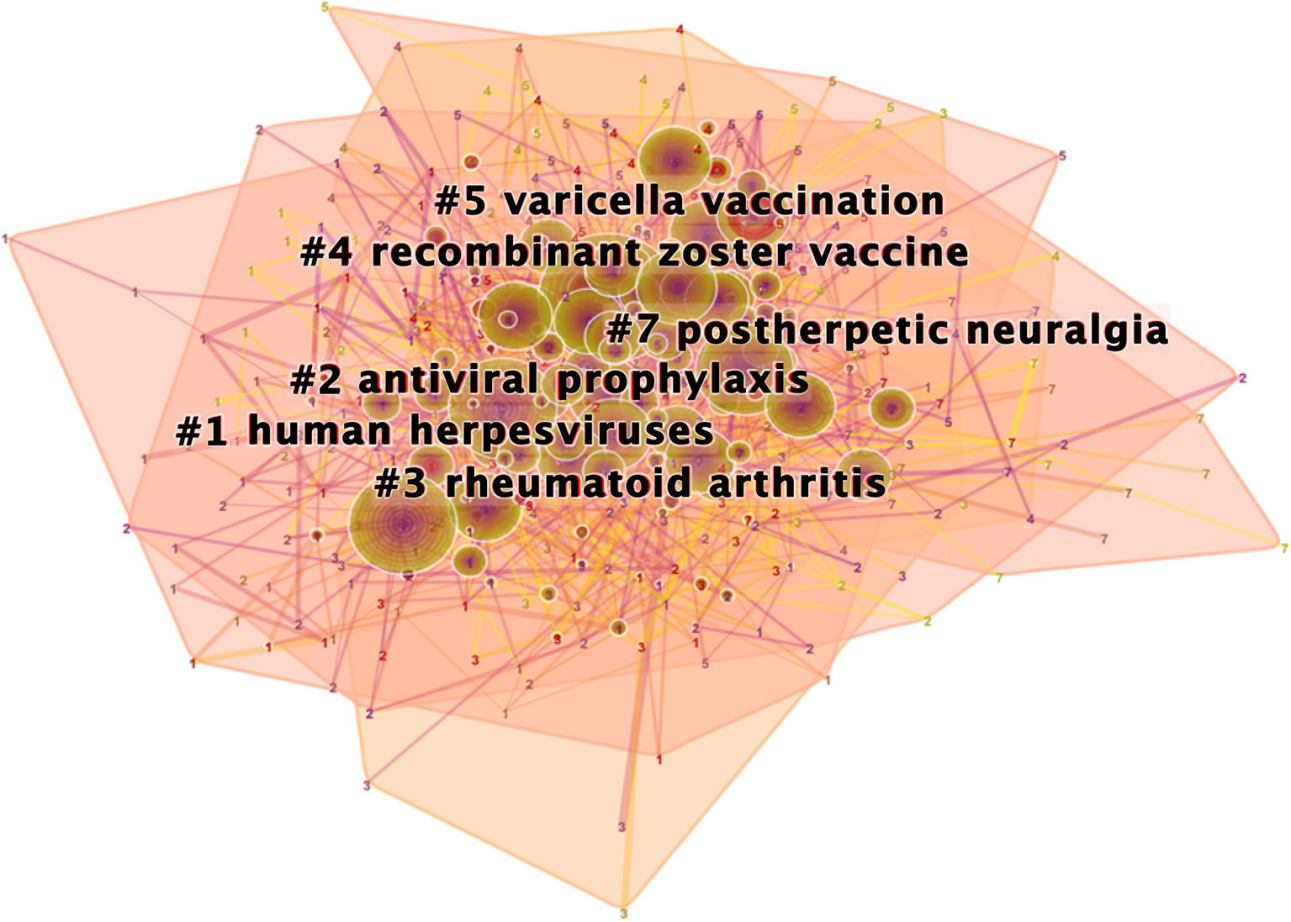
The clustered network map of keywords on herpes zoster.

**Figure 7:**
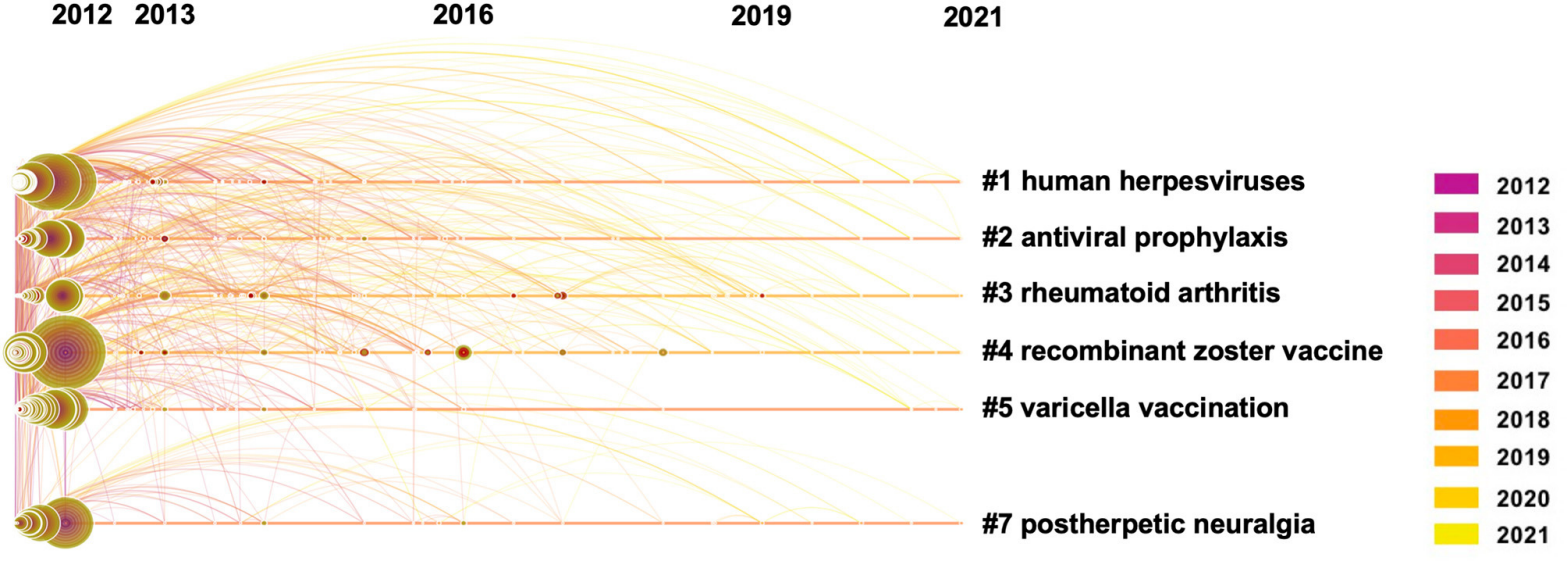
The timeline view of keyword clusters with their cluster-labels on the right.

Citespace revealed 571 uses of keywords from 2012 to 2021 on herpes zoster. Co-occurrence of the keywords suggested six clusters that represent the hot topics (Figure 6) and their timeline (Figure 7). Interestingly, rheumatoid arthritis was the third most frequent co-occurrence keyword (cluster 3); this may be related to the high incidence of banded blistering in patients with rheumatoid arthritis, relating to drugs used to treat rheumatoid arthritis (46). The other cluster labels were human herpesviruses, antiviral prophylaxis, recombinant zoster vaccine, varicella vaccination and postherpetic neuralgia. Moreover, herpes zoster (1,917), VZV (1,059), and infection (731) were the top-ranked frequent keywords, while mortality (0.06), resistance (0.06), and double blind (0.05) were the top-ranked central keywords of the analysis of co-occurrence of keywords (Table 6).

**Table 6:** Top 20 keywords in terms of frequency and centrality in Herpes zoster research.

**Ranking**	**Frequency**	**Keyword**	**Centrality**	**Keyword**
1	1,917	herpes zoster	0.06	mortality
2	1,059	varicella zoster virus	0.06	resistance
3	731	infection	0.05	double blind
4	615	postherpetic neuralgia	0.05	neuropathic pain
5	455	epidemiology	0.05	activation
6	396	efficacy	0.05	system
7	391	risk factor	0.05	simplex virus
8	386	herpes simplex virus	0.05	mutation
9	385	virus	0.04	reactivation
10	383	disease	0.04	varicella vaccine
11	368	risk	0.04	receptor
12	363	united states	0.04	age
13	358	children	0.04	angiotensin ii
14	337	expression	0.04	subthalamic nucleus
15	307	management	0.04	rat
16	286	diagnosis	0.04	inhibition
17	280	vaccine	0.04	viral infection
18	273	varicella-zoster virus	0.04	brain
19	272	safety	0.04	memory
20	252	therapy	0.03	risk

**Figure 8:**
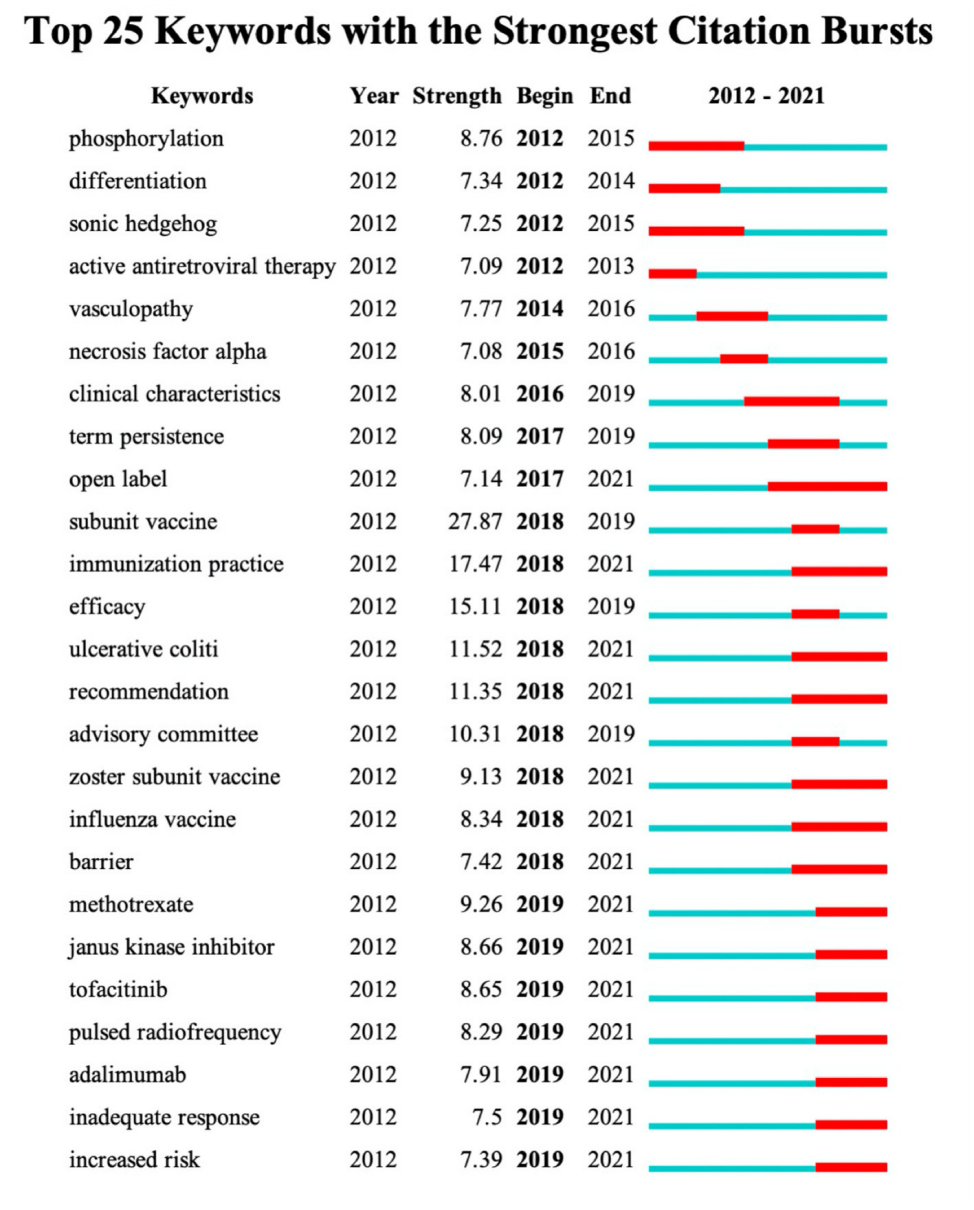
Keywords with the strongest citation bursts in publications on herpes zoster research between 2012 and 2021.

Burst keyword detection represent words that are cited frequently over a period of time (37). These are considered the indicators of frontier research topics over time. The bold red grid represents the time interval of the burst (Figure 8). The subunit vaccine had the highest burst strength (27.87), followed by immunization practice (17.47). Recent attention on subunit vaccine or zoster subunit vaccine from 2018, recommendation, immunization practice from 2018 till 2021 indicated that these are the current research hotspots.

## Discussion

This study visually analyzed a total of 9,259 herpes zoster publications (64.77% articles and 12.94% reviews) from 2012 to 2021 using bibliometrics methods. According to detailed analysis conducted using CiteSpace software, scientific research outputs concerning herpes zoster have clearly increased over the past 10 years. The U.S. was the leading country for both publications and centrality for its extensive international cooperation. The University of Colorado contributed the most publications, while the research of the Charite has the highest influence. The studies of representative authors and cited authors focus on vaccines, treatment, and mechanisms. The clusters of co-cited publications indicate that studies over the past 10 years are mainly focused on vaccines, postherpetic neuralgia, treatments, and VZV and its mechanism. Keyword analysis suggests that vaccines, especially subunit vaccines in different age groups, is the current hotspot in the field of herpes zoster.

Articles are the main type of herpes zoster publication. Unlike other topics, we can infer from the high frequency keywords that most of the research in this field is population-based or clinical trials. This is because VZV infection is highly restricted to humans. The development of a reliable animal model has been challenging, meaning that the understanding of VZV neurotropism in humans has long been hindered. For example, experimental inoculation of VZV in small animals, including guinea pigs and cotton rats, results in infection of the ganglia but not a rash (47). Only a few primate models, such as Simian varicella virus infection of rhesus macaques, closely resemble both human primary VZV infection and reactivation, with analyses at early times after infection providing valuable information about the extent of viral replication and the host immune responses (48). Therefore, most of these studies on herpes zoster are clinical applications.

As seen from the cooperation between countries, herpes zoster studies are concentrated in those countries with higher economic development, with the U.S. and Europe in the leading position. The People's Republic of China, Japan and South Korea are the only three countries from the Asian region in the top 10 collaborating countries. There is no doubt that the earlier initiation of research in this field in the U.S. and the Europe has led to the present trend. We believe that further progress will be made in the study of herpes zoster with the increasing attention of other countries and increased international cooperation and communication.

Another trend in herpes zoster research is the shift from symptomatic treatment to root treatment. In the early days, research mostly focused on the treatment of post-herpetic neuralgia. As mentioned earlier, pregabalin combined with neurotrophic drugs remains the most effective treatment, and early application of antiviral drugs after infection could reduce the proportion of post-herpetic neuralgia (6, 11). However, symptomatic treatment might lead to undesirable side effects such as rheumatoid arthritis (46, 49). The results from this bibliometric analysis suggest that vaccines might be the trend to treat herpes zoster at the root. The effectiveness of vaccines on preventing herpes zoster need further investigations. Increased understanding of the herpes zoster virus itself and the maturation of vaccine preparation techniques raise new hopes for the prevention of herpes zoster infection.

Vaccine research, especially in subunit vaccines, is the hotspot at the moment. “Subunit vaccine” is the representative burst keywords in recent years. The burst keywords were most clustered from highly cited articles or by conducting the most authoritative keyword analysis. It can be inferred that vaccine application in different age groups is at the forefront of research in this area. In fact, the efficacy and safety of vaccines for people over 50 have been widely validated and popularized (25, 38). Considerable progress has also been made in the development and application of vaccines for other age groups; for example, phase III trials have shown vaccines for children to be effective and safe (12, 22).

It is worth mentioning that COVID-19, an outbreak zoonotic disease affecting all mankind (50), can induce or worsen the symptoms of herpes zoster. As publications from 2020–2021 were included in this study, the clustered network map of co-cited references analysis reflected the relationship between COVID-19 infection and herpes zoster in cluster 20 (Figure 4). The emergence of latent infection with VZV may be affected by COVID-19, and the virus may induce reactivation of VZV in immunocompromised patients (51). In some cases, herpes zoster and severe acute herpetic neuralgia can be a complication of COVID-19 infection (52). Herpes can also be induced by vaccination against COVID-19, and in some cases, symptoms of herpes zoster have been reported in patients after vaccination (53–55).

This research has a number of limitations. First, the study design was based on publications included in the WoSCC database, and may therefore not include relevant publications in other sources such as Medline, SCOPUS, the Cochrane library, or Google Scholar. Literature searching with the same strategy got 9,136 and 8,553 publications in SCOPUS and Pubmed, relatively. As CiteSpace was developed based on WoS database, we selected WoSCC database for further study. Second, the CiteSpace analysis is based on the number of citations, but the number of citations is affected by many factors and cannot fully reflect the quality of the article. These limitations notwithstanding, we believe that this study is useful in understanding development trends and hot spots in this field, and guide the direction of further research.

## Conclusions

Herpes zoster has a high incidence and poor outcomes. Annual publications in this field have a steady increase in the past decade, with the U.S. accounting for most publications. Vaccines is the area of greatest interest for researchers. The keyword “subunit vaccine” indicates that a vaccine for herpes zoster prevention is now the direction of research and a research hotspot, as different populations (especially different age groups) have vaccine adaptability. This bibliometric study defines the overall prospects in the field of herpes zoster and provides valuable information for ongoing studies. Efforts to prevent VZV infection at source in different populations will be essential to improve the current poor outcomes associated with herpes zoster infection.

## Data Availability Statement

The original contributions presented in the study are included in the article/Supplementary Material, further inquiries can be directed to the corresponding author/s.

## Author Contributions

JZ and XH collected and analyzed the data. JZ, XH, DS, and XG wrote the manuscript. XG and WY designed the study and revised the manuscript. All authors contributed to the article and approved the submitted version.

## Funding

This work was supported by Shanghai Rising-Star Program (21QC1400300), National Natural Science Foundation of China (No.81701092, 81971223, 32030043), Shanghai Municipal Education Commission-Gaofeng Clinical Medicine Grant Support (20171916), Shanghai Engineering Research Center of Peri-operative Organ Support and Function Preservation (20DZ2254200), The Shanghai Pudong New Area Municipal Commission of Health and Family Planning Funding (PWZxq2017-06), Shanghai Municipal Key Clinical Specialty (shslczdzk03601).

## Conflict of Interest

The authors declare that the research was conducted in the absence of any commercial or financial relationships that could be construed as a potential conflict of interest.

## Publisher's Note

All claims expressed in this article are solely those of the authors and do not necessarily represent those of their affiliated organizations, or those of the publisher, the editors and the reviewers. Any product that may be evaluated in this article, or claim that may be made by its manufacturer, is not guaranteed or endorsed by the publisher.
