# Supplementary material for: Research Trends and Hotspots on Herpes Zoster: A 10-Year Bibliometric Analysis (2012–2021)
Source: Front Med (Lausanne). 2022 Apr 26;9:850762. doi: 10.3389/fmed.2022.850762 (PMC9089455; doi:10.3389/fmed.2022.850762)
Supplement: Supplementary file 1 [file Data_Sheet_1.DOCX]

**Supplementary Figures**


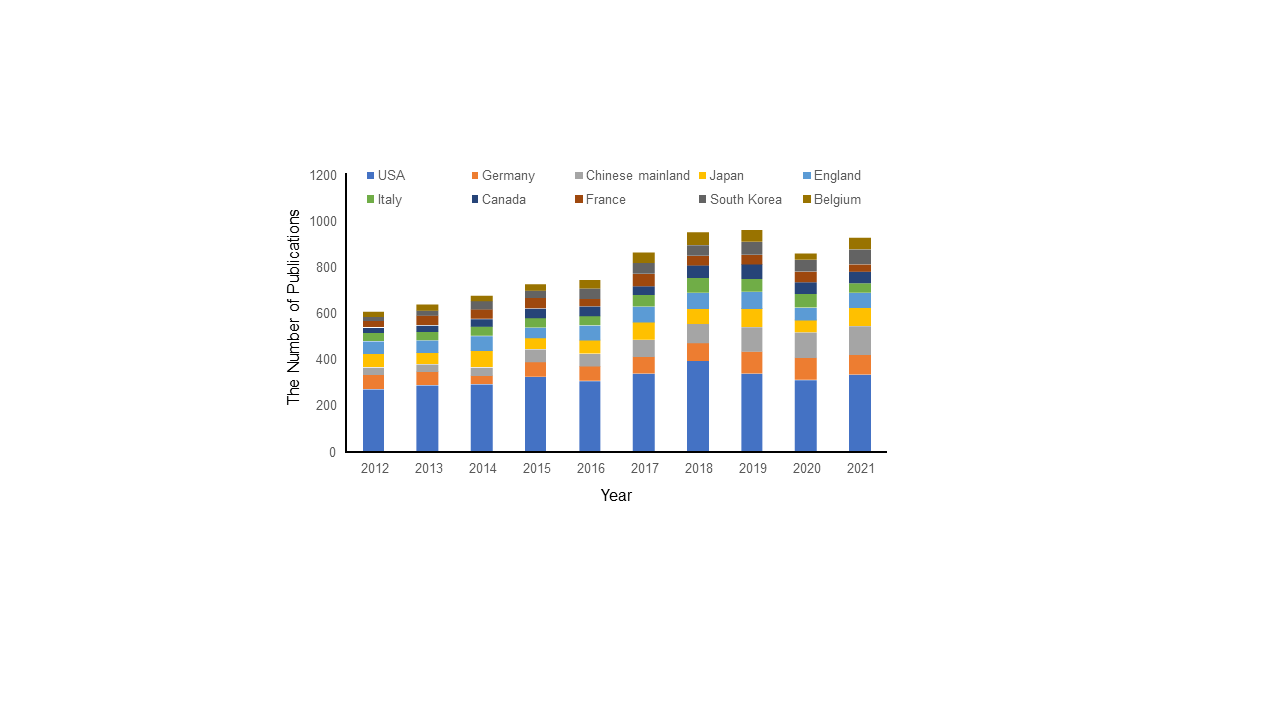


**Supplementary Figure 1.** The dynamic changes of the top 10 collaborating countries/regions in terms of publications in herpes zoster research.


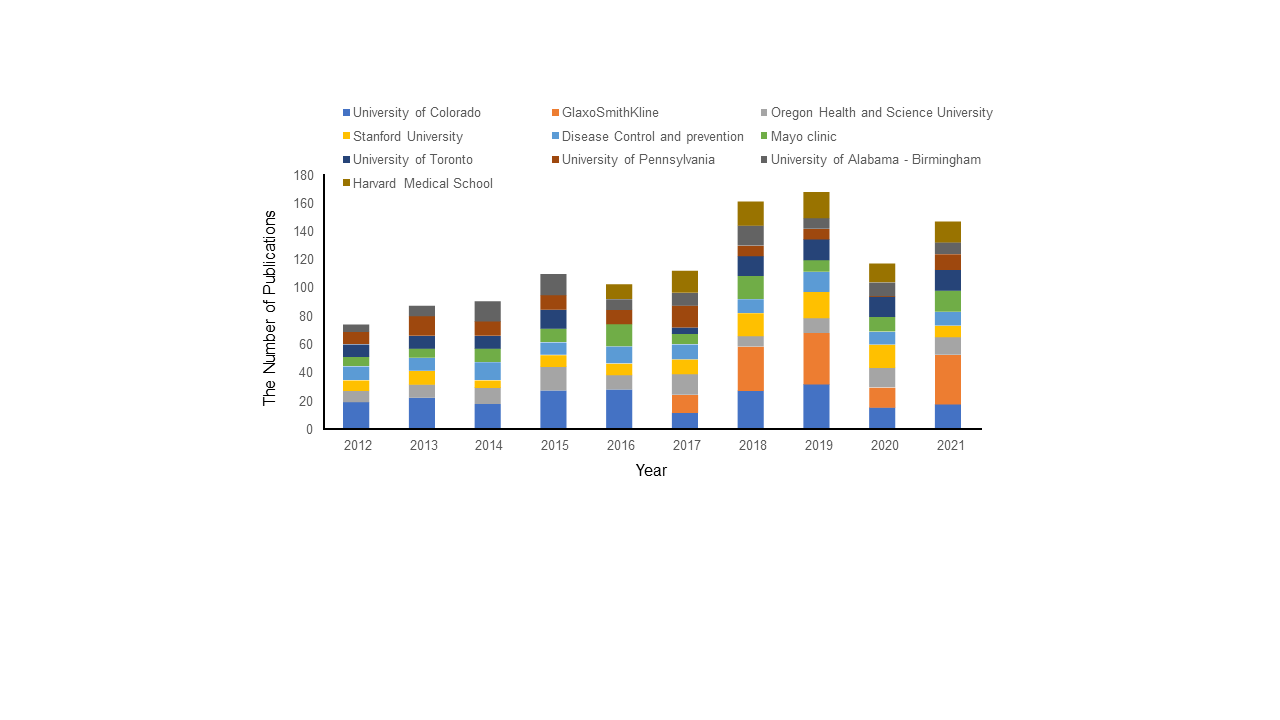


**Supplementary Figure 2.** The dynamic changes of the top 10 collaborating institutions in terms of publications in herpes zoster research.


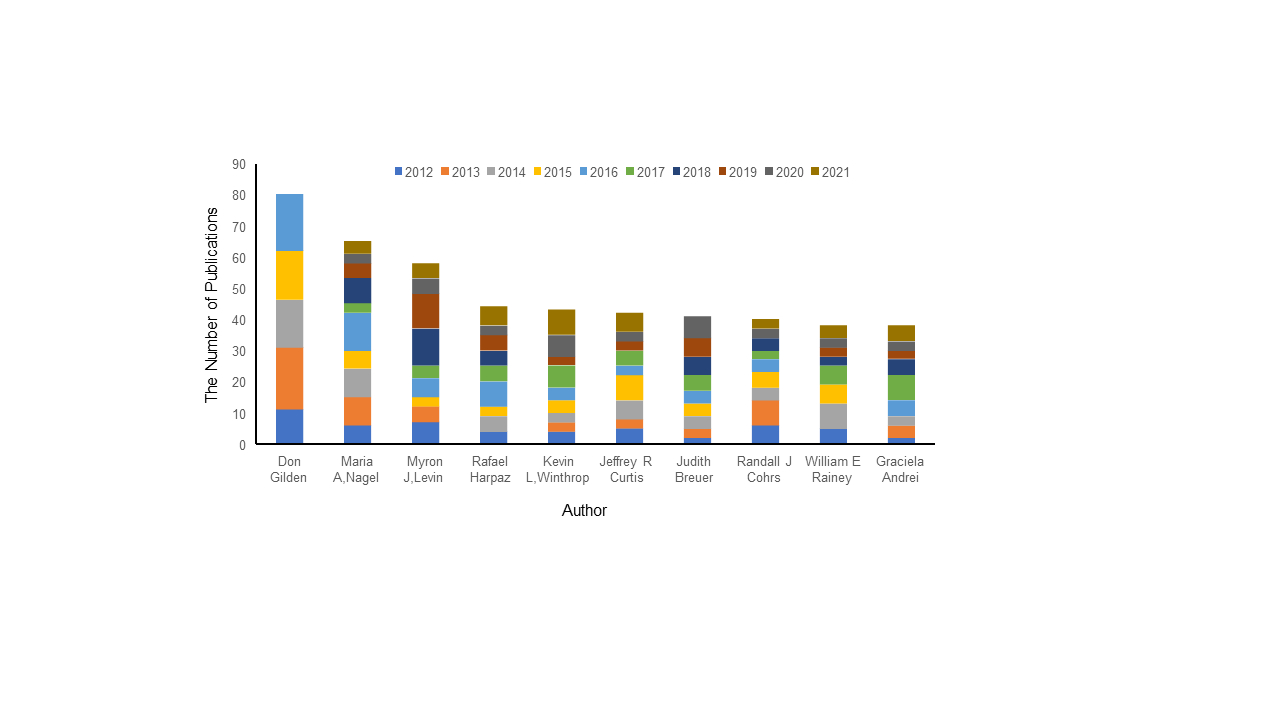


**Supplementary Figure 3.** The dynamic changes of the top 10 active authors in terms of publications in herpes zoster research.


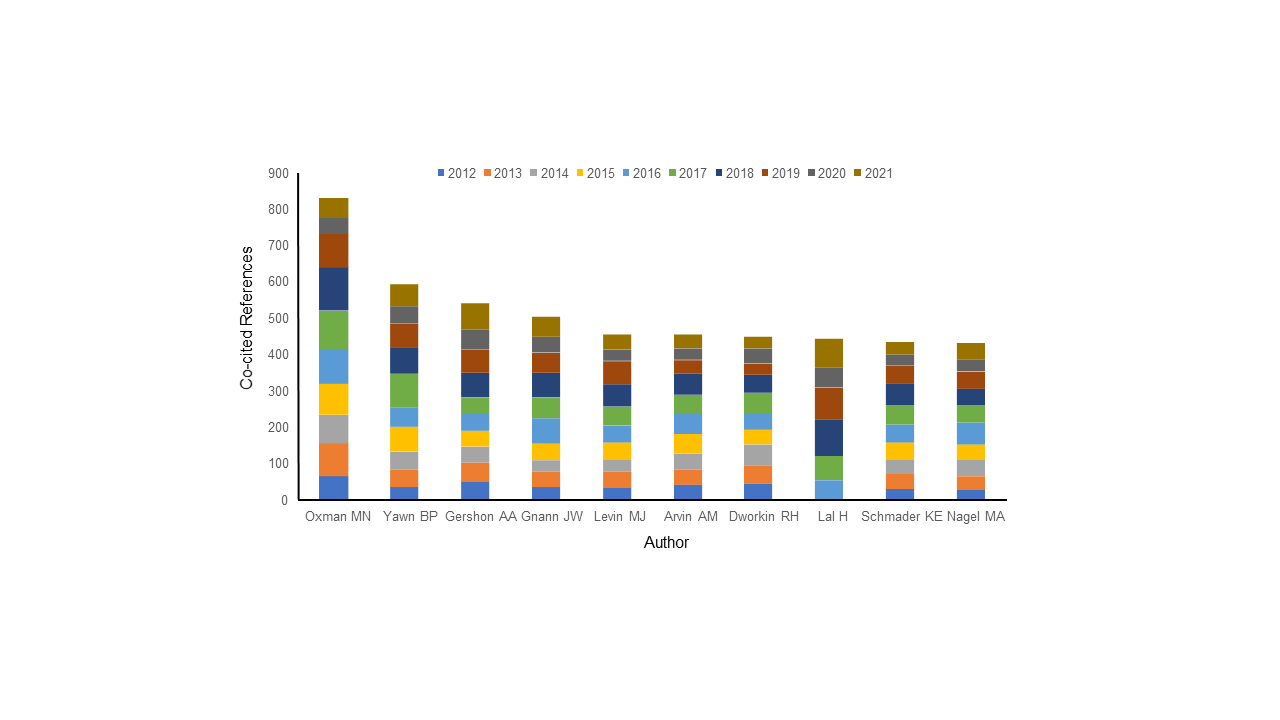


**Supplementary Figure 4.** The dynamic changes of the top 10 co-cited authors in herpes zoster research.
